# The prognostic value of vascular endothelial growth factor in 574 node-negative breast cancer patients who did not receive adjuvant systemic therapy

**DOI:** 10.1038/sj.bjc.6600555

**Published:** 2002-09-23

**Authors:** P Manders, L V A M Beex, V C G Tjan-Heijnen, J Geurts-Moespot, T H Van Tienoven, J A Foekens, C G J Sweep

**Affiliations:** Department of Medical Oncology, University Medical Centre Nijmegen, Nijmegen, The Netherlands; Department of Chemical Endocrinology, University Medical Centre Nijmegen, Nijmegen, The Netherlands; Department of Medical Oncology, Rotterdam Cancer Institute and University Hospital Rotterdam, Rotterdam, The Netherlands

**Keywords:** angiogenesis, node-negative breast cancer, prognostic value, vascular endothelial growth factor

## Abstract

The growth and metastasising capacity of solid tumours are dependent on angiogenesis. Vascular endothelial growth factor is a mediator of angiogenesis. In this study we investigated whether vascular endothelial growth factor is associated with the natural course of the disease in primary invasive breast cancer. In 574 tumours of patients with node-negative invasive breast cancer the cytosolic levels of vascular endothelial growth factor were measured using a quantitative enzyme-linked immunosorbent assay. These patients did not receive adjuvant systemic therapy and were followed for a median follow-up time of 61 months (range 2–155 months) after the primary diagnosis. Correlations with well-known prognostic factors, and univariate and multivariate survival analyses were performed. Vascular endothelial growth factor level was positively associated with age and tumour size (*P*=0.042 and *P*=0.029, respectively). In addition, vascular endothelial growth factor level was inversely, but weakly correlated with progesterone receptor levels (PgR) (*r_s_*=−0.090, *P*=0.035). A high vascular endothelial growth factor level (equal or above the median level of 0.53 ng mg^−1^ protein) predicted a reduced relapse-free survival and overall survival in the univariate survival rate analysis (for both *P*=0.005). In the multivariate analysis as well, vascular endothelial growth factor showed to be an independent predictor of poor relapse-free survival and overall survival (*P*=0.045 and *P*=0.029, respectively), in addition to age, tumour size and PgR. The results show that cytosolic levels of vascular endothelial growth factor in tumour tissue samples are independently indicative of prognosis for patients with node-negative breast cancer who were not treated with adjuvant systemic therapy. This implies that vascular endothelial growth factor is related with the natural course of breast cancer progression.

*British Journal of Cancer* (2002) **87**, 772–778. doi:10.1038/sj.bjc.6600555 www.bjcancer.com

© 2002 Cancer Research UK

## 

The growth of solid tumours and their metastatic spread is angiogenesis-dependent (Folkman, 1990, 1995). Angiogenesis that results in tumour microvascularity is an acknowledged early requirement for both tumour growth and dissemination (Ellis and Fidler, 1996). The change to the angiogenic phenotype may be due to the over-expression of a number of endothelial growth factors, such as vascular endothelial growth factor (VEGF) (Folkman, 1995). VEGF works as a principal mediator of normal and pathological angiogenesis (Ferrara and Davis-Smyth, 1997) and is secreted by a wide variety of cell types, including neutrophils, platelets and tumour cells (Senger *et al*, 1993; Amoroso *et al*, 1997; Taichman *et al*, 1997; Verheul *et al*, 1997). Furthermore, tumour associated stroma has also been shown to produce VEGF (Fukumura *et al*, 1998). VEGF consists of several splice variants yielding proteins of 121, 145, 165, 189, and 206 amino acids (Houck *et al*, 1991; Veikkola *et al*, 2000). In tissue, VEGF_165_ is the predominant isoform, and VEGF_121_ and VEGF_165_ are secreted into the circulation (Neufeld *et al*, 1999). Furthermore, related peptides have been described, i.e. VEGF-B, C, D and E (Neufeld *et al*, 1999).

Many types of malignant human tumours have been shown to produce VEGF. In previous studies the prognostic value of VEGF in patients with different malignancies, e.g. malignancies of the female tract (Abulafia *et al*, 1999; Hazelton *et al*, 1999; Santin *et al*, 1999; Tjalma *et al*, 2000; Boss *et al*, 2001), prostate (Bok *et al*, 2001), colon (Cascinu *et al*, 2001), urinary bladder (Sato *et al*, 1998; Crew *et al*, 1999), renal cell (Nicol *et al*, 1997) and thyroid gland (Lennard *et al*, 2000) has been investigated.

The prognostic value of VEGF has also been evaluated in invasive breast cancer in previous descriptive, retrospective studies (Gasparini *et al*, 1997, 2001; Eppenberger *et al*, 1998; Linderholm *et al*, 1998, 1999, 2000; Coradini *et al*, 2001). Two of these evaluated VEGF in heterogeneous series of patients including both node-negative and node-positive patients (Eppenberger *et al*, 1998; Linderholm *et al*, 2000). Five studies investigated the prognostic value of VEGF only in node-negative patients (Gasparini *et al*, 1997, 2001; Linderholm *et al*, 1998, 1999; Coradini *et al*, 2001). In three studies both treated and untreated patients were included (Eppenberger *et al*, 1998; Linderholm *et al*, 1998, 2000). Linderholm *et al* (1999) focused on the node-negative patients that were treated with radiotherapy. These previous studies have concluded that the concentration of VEGF was of prognostic value for relapse-free survival (RFS) (Gasparini *et al*, 1997, 2001; Eppenberger *et al*, 1998; Linderholm *et al*, 1999; Coradini *et al*, 2001) and/or overall survival (OS) (Gasparini *et al*, 1997; Linderholm *et al*, 1998, 1999, 2000) in different groups of patients.

In general, node-negative breast cancer patients who were treated with primary surgery have a relatively good prognosis, however, about 30% of these patients will develop distant metastasis within 10 years (Fisher *et al*, 1983; Saez *et al*, 1989). Prognostic factors are needed to separate node-negative patients into low-risk and high-risk groups in terms of the probability of recurrence and to focus the treatment efforts on patients at high risk. Importantly, to establish the prognostic value of a specific factor, the investigated patients should not have received adjuvant systemic therapy, because this may affect the relationship of the factor with the natural course of the disease. Three of the above mentioned studies included only patients who were not treated with adjuvant systemic therapy, but they did not take all established prognostic indicators into account in the performed analyses (Gasparini *et al*, 1997, 2001; Coradini *et al*, 2001). The principal aim of this study was to investigate the prognostic value of VEGF for RFS and OS in patients with node-negative breast cancer, who were not treated with adjuvant systemic therapy.

## PATIENTS AND METHODS

### Patients

A series of 1325 patients with operable breast cancer who underwent resection of their primary tumour between January 1987 and December 1996 were selected by the availability of frozen tissue in our tumour bank. This bank contains frozen tumour tissue of patients with breast cancer from nine different hospitals of the Comprehensive Cancer Center East in The Netherlands, because in this hospital (University Medical Centre, Nijmegen) the measurement of oestrogen receptor (ER) and progesterone receptor (PgR) levels, by means of the ligand-binding assay, was centrally done for these hospitals. The clinical data had to be retrospectively collected from these nine hospitals. To determine the prognostic value of VEGF, patients were selected by having node-negative breast cancer and by not having received adjuvant systemic therapy (*n*=666). Thirty-two patients had evidence of distant metastases at the time of diagnosis and eight had evidence of disease within 1 month after primary surgery. These patients were all excluded. Patients with previous diagnosis of carcinoma, with the exception of basal cell skin cancer were also excluded (*n*=20), as were patients with bilateral breast cancer (*n*=6) and patients with only carcinoma *in situ* (*n*=26). In total, 574 patients were considered assessable. Patients underwent a modified radical mastectomy (*n*=345) or a breast conserving lumpectomy with axillary lymph node dissection plus complementary radiotherapy (*n*=225). There were also four patients who were treated by breast conserving lumpectomy who did not receive additional radiotherapy. Of the patients who underwent a modified radical mastectomy, 64 received complementary radiotherapy. A resection was considered complete when there were no tumour cells in the inked border of the histological section. In case the margin was not free, a re-resection or breast ablation was performed whenever possible or additional radiotherapy was given. The median follow-up time was 61 months (range 2–155 months). The median age was 60 years (range 31–88 years). Further characteristics of patients and tumours are listed in Table 1

**Table 1:**
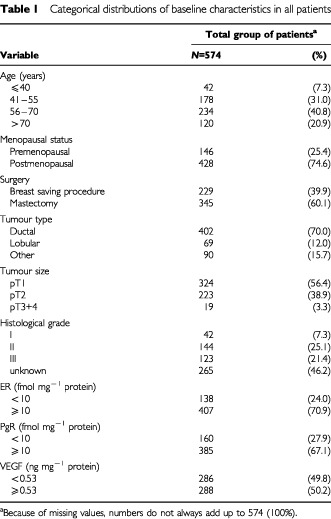
Categorical distributions of baseline characteristics in all patients

.

Of the 574 patients included in this study, 116 patients (20%) showed evidence of relapse of disease during follow-up. The first relapses observed were: local recurrence in 25 patients, distant metastasis in 77 patients and 14 patients had both. Forty-six patients died due to breast cancer, while 24 patients died without evidence of disease at last follow-up. There were 22 patients who had a secondary primary tumour after the primary breast tumour. These were not considered as failures.

### Tumour tissue processing

After the surgery, a representative part of the tumour was selected by the pathologist, frozen in liquid nitrogen and sent to this department (Department of Chemical Endocrinology). The primary breast cancer biopsies were stored in liquid nitrogen and pulverised in the frozen state with a microdismembrator as recommended by the European Organisation for Research and Treatment of Cancer (EORTC) for analysing ER and PgR (Eortc Breast Co-Operative group, 1980). The tissue powders were suspended in EORTC buffer, containing 20 mM K_2_HPO_4_/KH_2_PO_4_, 1.5 mM K_2_EDTA, 3 mM sodium azide, 10 mM monothioglycerol, 10% (v v^−1^) glycerol/water, pH 7.4 and centrifuged at 800 **g** for 20 min at 4°C. The supernatants were collected and subjected to further centrifugation for 1 h at 100 000 **g** (4°C). A part of the high speed supernatants obtained (cytosols) were used for measurement of ER and PgR levels by ligand-binding assay as previously described (Koenders *et al*, 1977), the remaining cytosols were stored at −80°C in liquid nitrogen. The protein concentrations were determined by the method of Lowry using BSA as the standard (Lowry *et al*, 1951).

### Vascular endothelial growth factor assay

VEGF levels were determined in the primary breast tumour cytosols with an ELISA developed by this department (Department of Chemical Endocrinology) for the Receptor Biomarker Group (RBG) of the EORTC. The assay measures VEGF_165_ and VEGF_121_, the main isoforms of VEGF. The details of the assay, including those of the specificity and performance, have been described elsewhere (Span *et al*, 2000).

To increase the sensitivity of the VEGF assay, the horseradish peroxidase labelled goat anti-rabbit detecting antibody was replaced by a goat anti-rabbit alkaline phosphatase conjugate (A-3687, Sigma Chemical Co, St. Louis, MO, USA). As substrate 4-methylumbelliferyl phosphate (M-6491, Molecular Probes, Eugene, OR, USA) in 10% diethanolamine was used. The reaction was stopped with 1 M NaOH. Fluorescence was measured with a fluorometric plate reader (Fluoroskan, Lab Systems, Oy, Helsinki, Finland) using 355 nm excitation and 460 nm emission filters.

### Data analysis

The median value of VEGF in this group of patients was used as the cut off value in the statistical analyses when analysing VEGF as a dichotomised variable.

To analyse interrelations between VEGF and various traditional parameters, Spearman rank correlations were calculated for continuous variables and the Kruskal–Wallis test for ordered variables.

Survival curves were generated using the method of Kaplan and Meier (1958). For the univariate survival rate analysis, RFS time (defined as the time from surgery until the diagnosis of recurrent disease) and OS time (defined as the time between date of surgery and death by any cause) were used as follow-up parameters. The survival curves only include the first 96 months of follow-up, because of the rapidly declining number of patients thereafter. Patients with events after 96 months were censored at 96 months

Cox univariate regression analysis was used in the analysis of the associations between the different variables and RFS and OS, and Cox multivariate regression analysis was used to evaluate the prognostic value of VEGF in addition to traditional factors (Cox, 1972). All computations were done with the SPSS statistical package (release 10.0.5, November 1999). Two-sided *P*-values below 0.05 were considered to be statistically significant.

## RESULTS

### Distribution of VEGF

Levels of VEGF protein were measured in 574 primary breast tumour cytosols. In this series, a wide range of concentrations of VEGF in cytosol, ranging from 0.00 to 48.03 ng mg^−1^ protein, was observed. The median cytosolic VEGF level was 0.53 ng mg^−1^ protein. This value was used as cut off value to enable the analysis of VEGF as a categorised variable (low, <0.53 ng mg^−1^ protein; high, ⩾0.53 ng mg^−1^ protein).

### Relationships

The tumour levels of VEGF were not related to those of ER (*r_s_*=−0.065, *P*=0.132), or associated with histological grade (*P*=0.082). Tumour VEGF levels were lower in patients with smaller, compared with those with larger tumours (*P*=0.029). Tumours from young patients had lower levels of VEGF than those of patients who were older (*P*=0.042). VEGF levels were higher in PgR-negative, compared with receptor-positive tumours (*r_s_*=−0.090, *P*=0.035).

### Survival rate analyses

The 5-year probability of RFS was 84% for patients with low VEGF levels and 75% for those with high VEGF levels. For OS, the 5-year probability was 93% for patients with a low VEGF level and 86% for those with a high level of VEGF.

Figure 1

**Figure 1:**
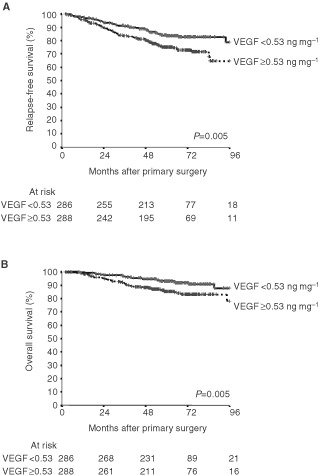
RFS (**A**) and OS (**B**) as a function of VEGF values. For each data set original VEGF values in the primary breast tumours were divided into two groups: < or ⩾0.53 ng mg^−1^ protein. Events indicate the number of patients at risk in each group. Patients at risk at 0, 24, 48, 72 and 96 months after primary surgery are indicated.

show the results of the univariate RFS and OS rate analyses for the total group of patients. After 8 years 79% of the patients of whom the tumour had low VEGF levels (<0.53 ng mg^−1^ protein) were recurrence-free compared with 65% of those with high VEGF levels (*P*=0.005). When VEGF was used as a continuous variable, also a significant relationship of higher VEGF levels with a poor RFS was observed (*P*=0.004). Similarly, high VEGF levels were associated with a poor OS, both when analysed as a continuous variable (*P*=0.004) and as a dichotomised variable (*P*=0.005). At 8 years, 88% of the patients were alive in the group of patients with low VEGF levels compared with 78% in patients with high VEGF levels (Figure 1).

### Cox analysis

In the Cox univariate regression analysis, young age, larger tumour size, and negative ER and PgR status were significantly associated with a poor RFS and OS (Table 2

**Table 2:**
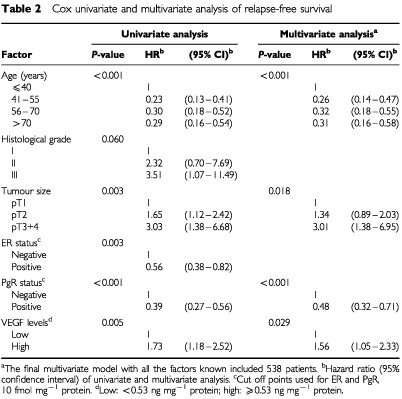
Cox univariate and multivariate analysis of relapse-free survival

and 3

**Table 3:**
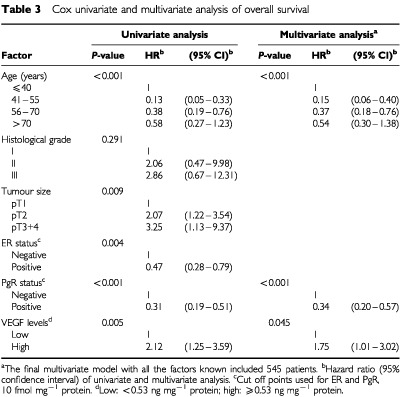
Cox univariate and multivariate analysis of overall survival

). In contrast, histological grade showed neither a significant association with RFS nor with OS. Since VEGF was shown to be of prognostic value in univariate analysis, Cox multivariate analysis was performed to evaluate whether it might significantly add to the contribution of the traditional prognostic factors.

In the final multivariate model, VEGF did still contribute to the prognostic information already provided by the traditional prognostic factors in the analysis for both RFS and OS (Table 2, RFS: HR=1.56, *P*=0.029; Table 3, OS: HR=1.75, *P*=0.045). Together with VEGF, age and PgR were significantly associated with both RFS and OS, while ER and histological grade were not. Tumour size was only significantly associated with RFS.

## DISCUSSION

In this retrospective study, the prognostic value of VEGF was evaluated in 574 node-negative patients with primary breast cancer. None of these patients were treated with adjuvant systemic therapy, thus enabling the study of VEGF in relation to the natural course of the disease. VEGF levels were found to be positively associated with tumour size and negatively associated with ER and PgR status. A high level of VEGF (⩾0.53 ng mg^−1^ protein) was shown to independently predict a short RFS and OS in patients with node-negative breast cancer, in addition to age, tumour size and PgR.

Of note, these results in fact support the conclusion reported by other investigators, i.e. that VEGF is indeed of prognostic significance in breast cancer (Gasparini *et al*, 1997, 2001; Eppenberger *et al*, 1998; Linderholm *et al*, 1998, 1999, 2000; Coradini *et al*, 2001). Three of the previous studies investigated the prognostic value of VEGF in node-negative patients who were not treated with adjuvant systemic therapy, which were similar to the current study (Gasparini *et al*, 1997, 2001; Coradini *et al*, 2001). Gasparini *et al* (1997) reported that VEGF was an independent prognostic factor of RFS and OS in multivariate analyses in a smaller group of node-negative patients with primary breast cancer (*n*=260), who were not treated with adjuvant systemic therapy. Unfortunately, they did not take age and histological grade into account in the multivariate analysis, although these are established prognostic indicators. In another study of Gasparini *et al* (2001) on the prognostic value of thrombospondins-1 and -2 and their correlation with VEGF and thymidine phosphorylase, VEGF was statistically significant for RFS in 168 node-negative patients, who were not treated with adjuvant systemic therapy, but again they did not take all established prognostic factors into account in the multivariate analysis, e.g. age, histological grade, ER and PgR. In a study of Coradini *et al* (2001) on the contribution of VEGF to the Nottingham prognostic index, VEGF was of borderline significance for RFS in 226 node-negative patients who were not treated with adjuvant systemic therapy.

Eppenberger *et al* (1998) showed that VEGF was prognostically significant for poor RFS in 305 node-negative and node-positive breast cancer patients. However, the investigators also included patients who received adjuvant systemic therapy, which makes it more difficult to draw conclusions about the true prognostic impact of VEGF. In the study of Linderholm *et al* (1998), VEGF was also statistically significantly associated with OS in the multivariate analysis in 525 node-negative patients, but again patients who had received adjuvant systemic therapy were included. Moreover, these authors only investigated the association with OS and not with RFS. In another study Linderholm *et al* (1999) reported that VEGF was of borderline significance for OS in the multivariate analysis in a small group of node-negative patients who were treated with radiotherapy. Linderholm *et al* (2000) investigated the prognostic value of p53 and VEGF in node-negative breast cancer patients. In this study VEGF was also statistically significantly associated with OS but not with RFS in the multivariate analysis (*n*=485), but again patients who had received adjuvant systemic therapy were included.

In the above mentioned studies, a clear distinction between the prognostic *vs* predictive value of a factor was not always made. In an earlier study by Foekens *et al* (2001) on the predictive value of VEGF in breast cancer patients with advanced disease, it was shown that a high VEGF level predicts a poor efficacy of both tamoxifen and chemotherapy in advanced breast cancer. It is important to stress, that to determine the prognostic value of a marker, patients who did not receive adjuvant systemic treatment should ideally be studied. In addition, the primary endpoint of such analyses should be RFS and not OS, as for the latter, the impact of systemic adjuvant treatment after relapse is also taken into account.

In the current study the histological grade is missing for 46% of the patients. In previous studies 20–25% of the patients had a missing value for this variable (Eppenberger *et al*, 1998; Linderholm *et al*, 1998, 1999, 2000) or it was not included in the analyses (Gasparini *et al*, 1997, 2001). Coradini *et al* (2001) included the Nottingham prognostic index, which is based on morphopathologic features, i.e. lymph node status, tumour size and histological grade. Also, in the current study, the histological grade was included in the univariate Cox analyses for those patients for which it was available, but probably due to a substantial number of missing data, it was not a significant predictor of relapse.

It was not the intention of this study to find an optimal cut off value for VEGF in cytosols of tumour tissue of breast cancer patients and it was decided to use the median level in the group of patients. From a biological point of view, such an arbitrary assignment might be inappropriate. Alternatively, every conceivable cut off value might be sequentially examined to maximise the separation of the RFS and OS curves. However, if the total data set would have been used to find the optimal cut off value, one still needs an independent data set to validate this cut off value (Mcguire, 1991). Further studies are needed to determine the optimal cut off value for clinical use. Two of the before mentioned studies also used the median value as the cut off value (Linderholm *et al*, 1998, 1999). Eppenberger *et al* (1998) used the first quartile value to dichotomise the sample set. On the other hand, Gasparini *et al* (1997, 2001) used a spline function and Linderholm *et al* (2000) identified the cut off value with the smallest *P*-value and the highest relative risk for death. Coradini *et al* (2001) did not dichotomise the sample set, they used the 25th, 50th and 75th percentiles for VEGF.

Several studies documented that in invasive breast cancer the level of VEGF is highly associated with the degree of angiogenesis, assessed by microvessel count (Toi *et al*, 1995b; Anan *et al*, 1996; Obermair *et al*, 1997). Previous investigators have studied the prognostic value of the degree of vascularisation of the tumour by measuring intratumoural microvessel density (MVD). Some of them demonstrated that MVD is of prognostic value for patients with node-negative primary breast cancer (Gasparini *et al*, 1994; Obermair *et al*, 1995; Toi *et al*, 1995a), but not all (Ellis and Fidler, 1995).

It is of clinical interest to establish new prognostic factors that, in addition to the factors used up till now, could distinguish subgroups of patients with node-negative invasive breast cancer with a high risk for relapse that might benefit from adjuvant systemic therapy (National Institutes of Health Consensus Development Panel, 1992; Henderson, 1992; Osborne, 1992; Gasparini *et al*, 1993). Knowledge of these factors is also important for stratification in phase III trials and to explain different outcomes between trials with a comparable design.

In conclusion, our data suggest that VEGF is an independent prognostic factor for RFS and OS in patients with node-negative breast cancer. The present findings indicate that VEGF represents a biologic marker of breast tumour angiogenesis. Further studies are warranted to investigate whether patients with high VEGF levels are more likely to gain benefit from (antiangiogenic) systemic adjuvant therapy.
